# Endothelial Activation and Stress Index as an Indicator of Early Hemodynamic Instability in Critically Ill Patients: A Single-Centre Observational Study

**DOI:** 10.3390/diagnostics16091355

**Published:** 2026-04-30

**Authors:** Mateusz Jerzy Kreczko, Maria Bieniaszewska, Karol P. Steckiewicz, Radosław Owczuk

**Affiliations:** 1 Department of Anesthesiology and Intensive Therapy, Faculty of Medicine, Medical University of Gdańsk, 80-210 Gdańsk, Poland; karol.steckiewicz@gumed.edu.pl (K.P.S.); radoslaw.owczuk@gumed.edu.pl (R.O.); 2 Department of Anesthesiology and Intensive Therapy, University Clinical Centre, 80-214 Gdańsk, Poland; 3 Department of Hematology and Transplantology, Faculty of Medicine, Medical University of Gdańsk, 80-210 Gdańsk, Poland; maria.bieniaszewska@gumed.edu.pl; 4 Department of Hematology and Transplantology, University Clinical Centre, 80-214 Gdańsk, Poland

**Keywords:** EASIX, modified EASIX, simplified EASIX, illness severity, intensive care unit, endothelial dysfunction

## Abstract

**Background**: The Endothelial Activation and Stress Index (EASIX) is a biomarker initially developed to predict survival in patients with acute graft-versus-host disease after allogeneic haemato-poietic stem cell transplantation and is regarded as a surrogate of endothelial dysfunction. This study aimed to evaluate whether EASIX reflects early hemodynamic instability and vasopressor requirement in critically ill patients. **Methods**: We retrospectively analysed 447 patients admitted to the intensive care unit (ICU) at the University Clinical Centre in Gdańsk. Illness severity scores—including the Simplified Acute Physiology Score II (SAPS II), Acute Physiology and Chronic Health Evaluation II (APACHE II), and Sequential Organ Failure Assessment (SOFA)—and laboratory parameters, were collected at admission. EASIX, simplified EASIX (sEASIX), and modified EASIX (mEASIX) were calculated using established formulas. Vasopressor requirements, ex-pressed as norepinephrine equivalents (NEE), were recorded during the first 72 h. Statistical analyses included Spearman’s correlation, logistic regression, and receiver operating characteristic curve analysis. **Results**: In univariate analysis, EASIX was associated with ICU mortality (OR 1.333; 95% CI 1.135–1.576), but this association was not significant after adjustment. EASIX positively correlated with vasopressor requirements, severity scores (SOFA, SAPS II, APACHE II), and inflammatory and metabolic markers (PCT, CRP, lactate). It correlated with norepinephrine-equivalent doses within the first 48 h and moderately discriminated high-dose vaso-pressor use (>0.1 µg/kg/min). A weak negative correlation with ICU length of stay was observed. No association with age was found. **Conclusions**: EASIX is an age-independent marker associated with disease severity and early vaso-pressor burden in ICU patients. Rather than providing a direct measurement of endothelial function, it reflects a global signal of systemic stress and microvascular derangement and should be interpreted accordingly.

## 1. Introduction

In critically ill patients, rapid and accurate prognosis assessment is important for therapeutic processes. General illness severity scores such as the Acute Physiology and Chronic Health Evaluation II (APACHE II), Simplified Acute Physiology Score II (SAPS II), Sequential Organ Failure Assessment (SOFA), and Multiple Organ Dysfunction Score (MODS) are widely used in the ICU to predict outcomes and characterise disease severity and degree of organ dysfunction [1,2]. Despite the established utility of these scoring systems, several limitations persist. These include computational complexity, a need for extensive data collection, the potential for missing values, and variable performance across different populations and patient disease states that do not fully reflect the dynamics of pathophysiological processes occurring in critically ill patients [2]. These limitations have prompted a focus on biomarkers reflecting specific underlying processes, particularly endothelial dysfunction, systemic inflammation, and derived inflammatory indices [3,4,5].

The endothelium, which is a monolayer of polarised cells that covers the inner wall of blood vessels, separates circulating blood cells from the surrounding tissues. The estimated total combined surface area of the endothelium in the human body is 4000–7000 m^2^ [6]. The endothelium functions as a polarised endocrine organ that regulates vascular constriction, relaxation, regional blood flow, and the permeability of fluids and cells [7,8]. The proper function of the endothelium is based on the difference in electrical load; the luminal membrane is directly exposed to blood constituents and circulating cells, whereas the basolateral surface is separated from surrounding tissues by a glycoprotein basement membrane [9]. Endothelial dysfunction significantly impacts complications and prognosis in ICU patients. In states of sepsis and SIRS, cytokine-mediated shedding of the endothelial glycocalyx leads to profound microvascular failure, characterised by increased permeability (third spacing) and reduced nitric oxide bioavailability. The resulting dysregulated vasomotor tone and pro-coagulant shift in the denuded endothelium promote microthrombi formation, further accelerating the progression of organ failure [9,10,11].

The availability of direct biomarkers of endothelial injury and activation in critically ill patients is a topic of research gaining traction and has included markers for glycocalyx degradation (such as syndecan-1 and heparan sulphate), biomarkers for endothelial activation (von Willebrand factor, soluble thrombomodulin, angiopoietin-2), and functional bedside assessment such as brachial flow-mediated dilation [12,13]. Conceptual frameworks such as the Shock-Induced Endotheliopathy (SHINE) paradigm have unified these observations across diverse acute critical illnesses [14]. Biomarkers of this kind are important but, as such, their clinical applications in the intensive care unit (ICU) are often hampered by high cost, a lack of availability of appropriate test suites, non-standardisation in their approaches, and a lack of the capacity to obtain a validated threshold for decision by any means [5]. Regarding this topic, composite indices obtained from lstanrad laboratory parameters (e.g., ESAIX) have generated increasing interest as useful bedside surrogates for microvascular and endothelial derangement, even if they do not measure endothelial function directly. The Endothelial Activation and Stress Index (EASIX) represents a novel biomarker first described in 2017 by Luft et al., developed to predict survival in patients with acute graft-versus-host disease [15]. It is calculated using three readily available laboratory parameters: lactate dehydrogenase (LDH) activity, creatinine concentration, and thrombocyte count. Each component represents a different facet of endothelial injury: LDH levels increase due to release from endothelial cells, platelets and leukocytes when the vascular endothelium is damaged [16,17]. High serum creatinine concentrations reflect the influence of renal endothelial dysfunction, which is a key pathophysiological process associated with impaired renal function [18]. Additionally, a low platelet count may also be partly attributable to endothelial injury and complement activation [19]. Increased levels of collagen, von Willebrand factor, and tissue factor caused by vascular endothelial damage promote platelet hyperactivation and hyperaggregation [20]. The combination of these parameters reflects the extent of endothelial activation and systemic injury [21]. Two modifications of the EASIX score were later implemented: the simplified EASIX (sEASIX) and the modified EASIX (mEASIX). Only LDH and platelet count are used to calculate sEASIX, and LDH levels, C-reactive protein (CRP) concentrations and platelet count are used to calculate mEASIX [22,23]. Although each component of EASIX has been linked, on biological grounds, to specific aspects of endothelial injury, the index itself does not measure endothelial function directly. Rather, it integrates a composite signal of tissue injury, renal microvascular impairment and platelet consumption, all of which may also be influenced by non-endothelial pathways. This indirect, composite nature should be kept in mind when interpreting EASIX in heterogeneous critically ill populations.

This study aimed to evaluate whether EASIX and its modifications reflect early hemodynamic instability and vasopressor requirements in a general ICU population.

## 2. Materials and Methods

This study was designed as an observational, retrospective, single-centre study conducted in Gdansk, Poland. The study protocol was approved by the Bioethics Committee for Scientific Research at the Medical University of Gdansk (NKBBN/485/2021). Clinical data from patients hospitalised in the ICU of the University Clinical Centre in Gdansk between January and December 2021 were analysed. As this was a retrospective observational study using existing clinical data, prospective trial registration was not applicable.

A total of 447 patients treated in the Department of Anaesthesiology and Intensive Therapy, University Clinical Centre, Gdansk, Poland, in 2021 were included in the study. We excluded only patients whose records had missing laboratory values because they were needed for calculations. Clinical data were collected at ICU admission and included demographic information, the Simplified Acute Physiology Score (SAPS), the Acute Physiology and Chronic Health Evaluation II (APACHE II) score, the Sequential Organ Failure Assessment (SOFA) score, and routine laboratory parameters such as complete blood count and creatinine, C-reactive protein (CRP), procalcitonin (PCT), and lactate dehydrogenase (LDH) levels. Vasopressor requirements, expressed as norepinephrine equivalents, were recorded at admission and after 6, 12, 24, 48 and 72 h of hospitalisation. EASIX, sEASIX and mEASIX were calculated on the basis of previously described formulas [15,22,23].

(1) $$EASIX=\frac{LDH\;\left[U\;L^{-1}\right]\times creatinine\;[mg\;{dL}^{-1}]}{platelet\;count\;[G\;L^{-1}]}\;$$

(2) $$sEASIX=\frac{LDH\;\left[U\;L^{-1}\right]\;}{platelet\;count\;[G\;L^{-1}]}$$

(3) $$mEASIX=\frac{LDH\;\left[U\;L^{-1}\right]\times CRP\;[mg\;{dL}^{-1}]}{platelet\;count\;[G\;L^{-1}]}$$

Norepinephrine equivalents were calculated according to the methods of Goradia et al. [24].

(4) $$NE=\frac{norepinephrine+epinephrine\;\;}{10}+\frac{dopamine}{100}+vasopressin\times 2.5$$

all in μg kg^−1^ min^−1^, except for vasopressin IU min^−1^.

### 2.1. Primary Outcome

The primary outcome was vasopressor requirement, expressed as norepinephrine equivalent dose exceeding 0.1 μg/kg/min. ICU mortality and length of stay were designated as secondary, descriptive endpoints. The six vasopressor assessment time points (admission, 6, 12, 24, 48, and 72 h) were defined a priori in the study protocol to capture the early dynamic trajectory of haemodynamic instability, which is clinically most relevant within the first 72 h of ICU admission.

### 2.2. Statistical Analysis

Values are expressed as numbers (percentages) or as medians with interquartile ranges [IQRs]. The normality of the data distribution was assessed using the D’Agostino–Pearson test and visual inspection of Q–Q plots. Comparisons between two groups were performed using the Mann–Whitney U test. Associations between variables were evaluated using Spearman’s rank correlation coefficient. Univariable relationships with the outcome were examined using simple logistic regression models. Multivariate logistic regression analysis included variables with *p* < 0.20 in the univariate analysis. Discriminative performance was evaluated using receiver operating characteristic (ROC) curve analysis. The area under the curve (AUC) was calculated with 95% confidence intervals.

Continuous predictors with skewed distributions were log-transformed using the formula log(X + 1) rather than raw values. This transformation was applied to mitigate right-skewness, stabilise variance, and improve model fit, whereas the addition of “+1” allowed the inclusion of zero values without producing undefined results [25]. Multivariate logistic regression for ICU mortality was included as an exploratory adjunct to the primary analyses and was not intended as a fully adjusted causal model. Comorbidity data were not systematically recorded in this retrospective dataset and could therefore not be incorporated as covariates. Subgroup analyses according to admission diagnosis were performed post hoc and were not pre-specified in the study protocol; their results should therefore be regarded as exploratory and hypothesis-generating rather than confirmatory.

All the statistical analyses were performed using GraphPad Prsim (version 11, GraphPad Software, Inc., San Diego, CA, USA). A two-sided *p* value < 0.05 was considered to indicate statistical significance.

The authors used an artificial intelligence-based language model (ChatGPT, OpenAI, GPT-5.3, accessed in 2026) solely to assist with language editing and improvement of clarity.

## 3. Results

All consecutive patients admitted to the ICU between January and December 2021 were screened for inclusion. The only exclusion criterion was the absence of one or more laboratory values required for EASIX, sEASIX, or mEASIX calculation (LDH, creatinine, platelet count, or CRP). No further inclusion or exclusion criteria were applied, as the study was designed to reflect a real-world, unselected general ICU population. A total of 447 patient records were reviewed, and their demographic and clinical characteristics are summarised in Table 1. Thirty-five records were excluded because of incomplete laboratory data necessary for the intended analyses. The most common reason for exclusion was the absence of a valid LDH measurement at admission, most commonly due to sample haemolysis or incomplete laboratory workup under emergency conditions; since LDH is an indispensable component of all three EASIX formulae, exclusion of these patients was analytically unavoidable. Comparison of available clinical parameters between excluded and included patients demonstrated comparable severity profiles: median age 61 years [IQR 46–71] vs. 61 years [IQR 44–71], SOFA score 11 [IQR 7.5–13] vs. 9 [IQR 7–11], APACHE II 19 [IQR 13–25] vs. 18 [IQR 12–25], and SAPS II 42 [IQR 38–55] vs. 41 [IQR 31–55], suggesting that the exclusion did not introduce substantial selection bias.

**Figure 1:**
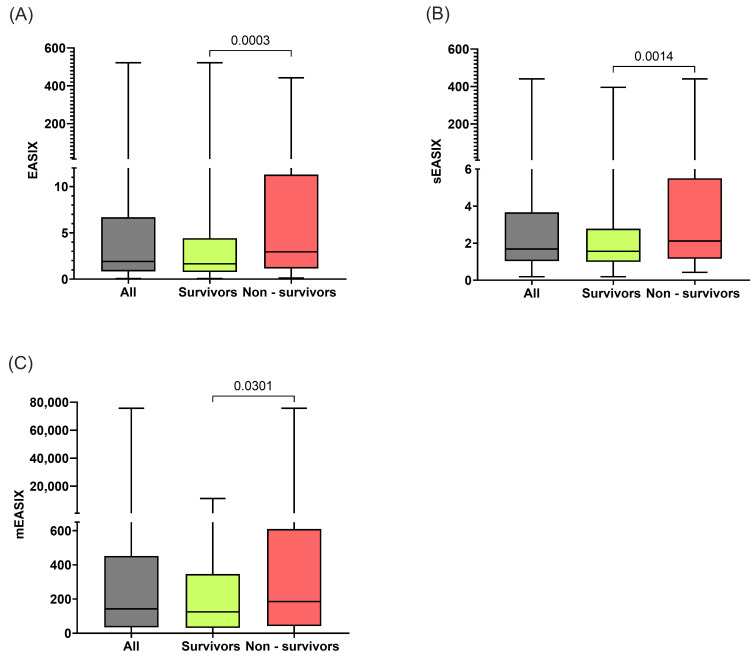
Distribution of (**A**) EASIX, (**B**) sEASIX, and (**C**) mEASIX values in survivors and nonsurvivors. The Y-axis is scaled unequally to enhance data visualisation, this does not affect statistical comparisons, which were performed on raw values. *p* values were calculated using the Mann–Whitney U test.

The distributions of EASIX, sEASIX, and mEASIX in the study population, as well as the differences between survivors and nonsurvivors, are presented in Figure 1. The median EASIX was 1.91 (IQR 0.84–6.69), the median sEASIX was 1.687 (IQR 1.03–3.67), and the median mEASIX was 142.2 (IQR 34.18–452.30). All three indices significantly differed between survivors and nonsurvivors (Figure 1).

Univariate analysis revealed that the EASIX, sEASIX, and mEASIX were risk factors for death during ICU stay (Table 2). Notably, the OR for sEASIX was the highest, whereas the EASIX had the tightest 95% CI for the OR. Multivariate analysis (including EASIX, SOFA, lactate) revealed that the EASIX was not an independent risk factor for death (OR 0.621; 95% CI 0.351–1.100).

Further analyses revealed positive correlations between the EASIX score and cumulative vasopressor dose, physiological scores (SOFA, SAPS II, and APACHE II) and biochemical parameters (PCT, CRP, and lactate concentrations). EASIX was not correlated with age. There was a weak negative correlation between the EASIX score and ICU length of stay. Interestingly, the EASIX score, which was calculated at admission, was positively correlated with norepinephrine-equivalent doses up to 48 h of hospitalisation (Table 3).

Subgroup analyses stratified by admission diagnosis revealed heterogeneity in the association between EASIX and vasopressor requirements across diagnostic categories (Table 4). In patients admitted following trauma, EASIX demonstrated the strongest correlations with norepinephrine-equivalent doses at admission and across the first 24 h (Spearman r = 0.53–0.56 at admission, remaining significant through 24 h). Similarly, in patients admitted for post-cardiac arrest care, significant correlations were observed from admission through 24 h (r = 0.38–0.43). In patients with respiratory failure, correlations were significant at admission and up to 12 h but attenuated thereafter. In contrast, no significant correlations between EASIX and vasopressor requirements were observed at any time point in patients admitted with sepsis.

EASIX demonstrated moderate discrimination for predicting vasopressor requirements (NEE > 0.1 μg/kg/min), with an AUC of 0.704 (95% CI 0.653–0.754) at admission. Its performance remained similar at 6 h (AUC 0.680) and 12 h (AUC 0.699), followed by a decline at 24 h (AUC 0.632) and 48 h (AUC 0.557). SOFA showed the highest predictive accuracy at all time points (AUC 0.786 at admission, decreasing to 0.607 at 48 h). In logistic regression analysis, each doubling of EASIX was associated with a 28% increase in the odds of high-dose vasopressor requirement at 6 h (OR 1.28, 95% CI 1.17–1.41, *p* < 0.001). APACHE II and SAPS II demonstrated lower and relatively stable discrimination (admission AUCs 0.623 and 0.632, respectively) (Table 5). Importantly, the cardiovascular component of SOFA directly incorporates the norepinephrine dose, while APACHE II includes mean arterial pressure (MAP) and SAPS II systolic blood pressure (SBP). In contrast, EASIX does not contain any hemodynamic variables.

It should be noted that the inclusion of SOFA in the comparative ROC analysis introduces conceptual circularity, as its cardiovascular component directly incorporates the norepinephrine dose. SOFA was therefore included not as an adjusted confounder but as a clinical benchmark, with the explicit purpose of contextualising EASIX performance. Crucially, EASIX contains no haemodynamic variables, and its moderate discriminative ability for vasopressor requirement reflects an independent, pathophysiological dimension of endothelial dysfunction rather than a derivative of circulatory parameters

## 4. Discussion

In this mixed ICU cohort, we found that higher EASIX, sEASIX, and mEASIX scores at admission were associated with increased ICU mortality. However, in the multivariate analysis, EASIX did not emerge as an independent risk factor for death. Key findings include a significant positive correlation between the admission EASIX score and cumulative vasopressor doses (norepinephrine equivalents) during the first 48 h of hospitalisation. To our knowledge, we are the first to report the relationship between EASIX score and vasopressor requirements in an ICU population.

The rationale for analysing vasopressor requirements at multiple time points (0–72 h) was to evaluate the performance of EASIX across the dynamic early phase of critical illness, where hemodynamic instability is most volatile. This longitudinal approach allowed us to demonstrate that EASIX correlates with vasopressor burden up to 48 h, highlighting its utility as an early indicator of sustained circulatory failure. While we acknowledge that a single measurement at admission creates a potential temporal mismatch with evolving outcomes, our results suggest that the initial degree of endothelial stress at ICU entry may predetermine the hemodynamic trajectory during the 48 h window. This temporal pattern is biologically expected: a single baseline measurement captures the degree of endothelial and systemic stress at one specific point of an evolving disease trajectory, after which therapeutic interventions (fluid resuscitation, vasopressors, source control, mechanical support) and the natural evolution of critical illness progressively dissociate the initial signal from the ongoing haemodynamic state [14,26]. From a clinical standpoint, this implies that admission EASIX is best positioned as an early triage and risk-stratification tool, rather than as a marker of subsequent treatment response or as a guide for therapy titration. Translating EASIX into actionable bedside use will therefore require studies evaluating serial measurements (e.g., at 24, 48 and 72 h) and their delta over time, in order to determine whether EASIX dynamics, rather than a single admission value, can capture evolving endothelial injury and inform decisions on escalation or de-escalation of haemodynamic support [27].

For NEE, commonly used cut-offs in the literature include 0.1, 0.25, and 1 µg/kg/min for mortality prediction and subgroup analyses [28]. The threshold of >0.1 μg/kg/min, was selected as a clinically established marker of significant hemodynamic instability, distinguishing between minimal support and high-dose requirements. This threshold is consistent with previous studies investigating endothelial dysfunction and its impact on macrocirculatory failure. Prior studies have demonstrated that NEE values around 0.1 µg/kg/min are associated with an increased mortality risk [29,30], while higher thresholds (≥0.2 µg/kg/min) are commonly used to define severe or refractory shock [31]. In addition, in another cohort (cardiac surgery patients) the median maximal dose of norepinephrine was 0.11 µg/kg/min [32]. These findings support the use of intermediate thresholds, such as 0.1 µg/kg/min, as clinically meaningful cut-offs reflecting moderate vasopressor dependency.

EASIX demonstrated moderate discrimination for predicting high-dose vasopressor requirements (>0.1 μg/kg/min) at admission and through the first 48 h. Our results extend the validated utility of EASIX—previously established in settings such as haematology, COVID-19, and sepsis to a general ICU population [15,33,34]. Previous studies have demonstrated that increasing EASIX values are associated with higher mortality risk, in different clinical settings [33,35,36]. Similarly, Luft et al. [37] reported that the EASIX score at hospital admission was a powerful predictor of severe COVID-19 (mechanical ventilation requirement and/or death) and mortality. In patients with haematologic malignancies, high EASIX was associated with worse outcomes; for example, Park et al. [38] reported that an EASIX score above the median predicted lower one-year survival in diffuse large B-cell lymphoma patients. The observed lack of correlation between EASIX and age suggests the index encapsulates acute pathophysiological disturbances rather than chronic frailty [33]. Additionally, the correlation between EASIX and markers of shock, such as lactate, underscores its role as a surrogate for microangiopathic stress, consistent with observations in other high-risk populations. This makes EASIX a pragmatic, bedside-ready tool for identifying patients at risk of early hemodynamic instability, even if it provides a functional rather than a mechanistic assessment of the endothelium.

The primary clinical value of EASIX lies in its reflection of a specific pathophysiological dimension endothelial and microvascular derangement without the inclusion of hemodynamic variables. In patients with sepsis and ARDS (including severe COVID-19), endothelial activation and injury are central to pathogenesis, causing capillary leakage, microthrombi and organ hypoperfusion [33,34]. While established scores like SOFA, APACHE II, and SAPS II demonstrate high predictive accuracy, they directly incorporate blood pressure or vasopressor doses into their algorithms. In contrast, EASIX relies solely on routine laboratory parameters: LDH (tissue injury/haemolysis), creatinine (renal microvascular impairment), and platelet count (consumption/microcirculation dysfunction) [15]. This makes it a pragmatic, bedside-ready tool for identifying patients at risk of early hemodynamic instability and intensified vasopressor support, aligning with modern frameworks for fluid and hemodynamic optimisation.

Subgroup analyses revealed that the association between EASIX and vasopressor requirements was strongest in trauma and post-cardiac arrest patients, and weakest in sepsis. This heterogeneity is biologically plausible. In trauma and post-cardiac arrest care, endothelial injury is predominantly acute and triggered by a single discrete event (mechanical disruption or global ischaemia–reperfusion) resulting in a tight coupling between admission EASIX and early haemodynamic instability [14,39]. In sepsis, however, endothelial dysfunction is a dynamic, multifactorial process modulated by cytokine cascades, fluid resuscitation, and antimicrobial therapy, which may attenuate the prognostic signal of a single admission measurement [40,41,42]. These findings suggest that EASIX may be most informative as an early haemodynamic predictor in non-infectious acute illness, and that its utility in sepsis warrants dedicated prospective evaluation. However, these findings were obtained from post hoc analyses and should be interpreted as hypothesis-generating. Although biologically plausible, they require confirmation in prospectively designed cohorts.

This study has several limitations, including its retrospective, single-centre design and a heterogeneous case mix which may limit generalizability. Heterogeneity likely diluted the independent prognostic signal of EASIX, as its components may be influenced by diverse non-endothelial pathways across different diagnostic categories. Furthermore, EASIX was only measured at admission, preventing the evaluation of temporal dynamics. Additionally, 35 patients were excluded due to missing LDH values, which precluded EASIX calculation; although the excluded patients showed comparable illness severity scores to the analytical cohort, the pattern of missingness, driven by haemolysis or incomplete sampling at admission, cannot be formally classified as missing completely at random. Comorbidity data were not available in this retrospective dataset and could not be incorporated into adjusted models. The multivariate mortality model relied on univariate *p*-value screening for variable selection and should therefore be interpreted as exploratory. The modelling strategy adopted in this study is intentionally simple. Therefore, residual confounding cannot be excluded, and the reported odds ratios and correlation coefficients should be interpreted as exploratory effect estimates rather than as adjusted causal effects. Finally, the absence of an external validation cohort and direct endothelial biomarker measurements means that prognostic thresholds require confirmation in prospective multicentre studies.

## 5. Conclusions

Our analysis identifies EASIX as a useful bedside indicator associated with microvascular derangement and early haemodynamic instability in critically ill patients. Rather than serving as a substitute for comprehensive scoring systems, it should be integrated as a complementary tool within clinical risk assessment frameworks. Future research should focus on mechanistic studies linking EASIX fluctuations to direct endothelial biomarker profiles. Additionally, prospective trials are needed to evaluate whether monitoring EASIX dynamics can guide therapeutic interventions or improve risk stratification in real-world, high-risk populations.

## Figures and Tables

**Table 1:** Population characteristics. Values are presented as numbers (%) or medians [IQRs].

Characteristics	Description
Patients	*N* = 447
Female	*N* = 133 (29.8%)
Age (yrs)	61.0 (44.0–70.8)
Main cause of ICU admission	Respiratory failure: *N* = 185 (41.4%) Trauma: *N* = 92 (20.6%) Sepsis: *N* = 92 (20.6%) Post-sudden cardiac arrest care: *N* = 78 (17.5%)
Treatment outcome	Death: *N* = 206 (46.1%) Discharge: *N* = 241 (53.9%)
Treatment length (days)	8 (4–16)
SAPS II score	41 (31–55)
APACHE II score	18 (12–25)
SOFA score	9 (7–11)
Lactate (mmol L^−1^)	1.4 (1.0–3.0)
Lactate dehydrogenase (U L^−1^)	334.0 (244.5–594.5)
Creatinine (µmol L^−1^) (mg dL^−1^)	92.82 (61.88–176.8) 1.05 (0.7–2.0)
C reactive protein (mg L^−1^) (mg dL^−1^)	915 (272–1914) 91.5 (27.2–191.4)
Procalcitonin (µg L^−1^)	1.4 (0.2–8.6)
Platelet count (×10^9^/L)	206 (147–285)

ICU—Intensive Care Unit; SAPS II—Simplified Acute Physiology Score II; APACHE II—Acute Physiology and Chronic Health Evaluation II; SOFA—Sequential Organ Failure Assessment; yrs—years.

**Table 2:** Factors influencing the risk of death during hospitalisation in the ICU (univariate analysis).

Parameter	OR	95% CI for OR	*p* Value
EASIX	1.333	1.135 to 1.576	0.0004
sEASIX	1.516	1.208 to 1.932	0.0003
mEASIX	1.286	1.015 to 1.639	0.0373

**Table 3:** Correlation of log(EASIX + 1) with other parameters.

Parameter	Spearman r	95% CI	*p* Value
Norepinephrine dose at admission	0.3415	0.2515 to 0.4255	<0.0001
Norepinephrine equivalents at admission	0.3425	0.2526 to 0.4265	<0.0001
Norepinephrine equivalents at 6 h	0.3761	0.2911 to 0.4552	<0.0001
Norepinephrine equivalents at 12 h	0.3492	0.2625 to 0.4303	<0.0001
Norepinephrine equivalents at 24 h	0.2509	0.1592 to 0.3383	<0.0002
Norepinephrine equivalents at 48 h	0.1190	0.0238 to 0.2120	0.0118
Norepinephrine equivalents at 72 h	−0.0007	−0.0962 to 0.0948	0.9884
ICU length of stay	−0.1861	−0.2804 to −0.0883	0.0001
SOFA at admission	0.5790	0.5087 to 0.6417	<0.0001
SAPS II	0.3673	0.2736 to 0.4542	<0.0001
APACHE II	0.3726	0.2736 to 0.4542	<0.0001
Procalcitonin	0.5605	0.4817 to 0.6303	<0.0001
C reactive protein	0.1527	0.05062 to 0.2516	0.0027
Lactate	0.4514	0.3692 to 0.5318	<0.0001
Age	0.07796	−0.02165 to 0.1760	0.1141

ICU—Intensive Care Unit; SAPS II—Simplified Acute Physiology Score II; APACHE II—Acute Physiology and Chronic Health Evaluation II; SOFA—Sequential Organ Failure Assessment.

**Table 4:** Correlation of log(EASIX + 1) with other parameters in subgroups.

Subgroup	Parameter	Spearman r	95% CI	*p* Value
Respiratory failure	Norepinephrine dose at admission	0.2737	0.1336 to 0.4030	0.0001
	Norepinephrine equivalents at admission	0.2679	0.1275 to 0.3977	0.0002
	Norepinephrine equivalents at 6 h	0.2756	0.1357 to 0.4048	0.0001
	Norepinephrine equivalents at 12 h	0.2157	0.07260 to 0.3501	0.0026
	Norepinephrine equivalents at 24 h	0.06640	−0.07973 to 0.2097	0.3589
	Norepinephrine equivalents at 48 h	−0.03384	−0.1783 to 0.1121	0.6403
	Norepinephrine equivalents at 72 h	−0.09976	−0.2416 to 0.04627	0.1675
Trauma	Norepinephrine dose at admission	0.5277	0.3438 to 0.6725	<0.0001
	Norepinephrine equivalents at admission	0.5590	0.3824 to 0.6962	<0.0001
	Norepinephrine equivalents at 6 h	0.4669	0.2707 to 0.6259	<0.0001
	Norepinephrine equivalents at 12 h	0.4557	0.2574 to 0.6171	<0.0001
	Norepinephrine equivalents at 24 h	0.4586	0.2609 to 0.6194	<0.0001
	Norepinephrine equivalents at 48 h	0.1706	−0.05619 to 0.3806	0.1279
	Norepinephrine equivalents at 72 h	−0.04334	−0.2653 to 0.1830	0.7008
Sepsis	Norepinephrine dose at admission	0.1447	−0.09869 to 0.3717	0.2287
	Norepinephrine equivalents at admission	0.1526	−0.09067 to 0.3787	0.2040
	Norepinephrine equivalents at 6 h	0.2218	−0.01911 to 0.4384	0.0630
	Norepinephrine equivalents at 12 h	0.1747	−0.06808 to 0.3980	0.1451
	Norepinephrine equivalents at 24 h	−0.01157	−0.2508 to 0.2290	0.9237
	Norepinephrine equivalents at 48 h	−0.1331	−0.3615 to 0.1104	0.2686
	Norepinephrine equivalents at 72 h	−0.08453	−0.3180 to 0.1586	0.4834
Post resuscitation care	Norepinephrine dose at admission	0.3762	0.1425 to 0.5703	0.0017
	Norepinephrine equivalents at admission	0.3826	0.1497 to 0.5753	0.0014
	Norepinephrine equivalents at 6 h	0.4321	0.2072 to 0.6136	0.0003
	Norepinephrine equivalents at 12 h	0.3773	0.1437 to 0.5711	0.0016
	Norepinephrine equivalents at 24 h	0.3288	0.08901 to 0.5326	0.0066
	Norepinephrine equivalents at 48 h	0.1328	−0.1181 to 0.3677	0.2842
	Norepinephrine equivalents at 72 h	−0.02804	−0.2732 to 0.2205	0.8218

**Table 5:** AUROC for prediction of vasopressor requirement (norepinephrine equivalents (NEE) > 0.1 µg/kg/min). AUC [95% CI of AUC].

	NEE at Admission	NEE at 6 h	NEE at 12 h	NEE at 24 h	NEE at 48 h
Log(EASIX + 1)	0.704 [0.653–0.754]	0.680 [0.628–0.731]	0.699 [0.616–0.722]	0.632 [0.580–0.688]	0.557 [0.495–0.618]
SOFA at admission	0.786 [0.742–0.828]	0.741 [0.695–0.789]	0.684 [0.649–0.746]	0.647 [0.599–0.699]	0.607 [0.555–0.662]
APACHE II	0.623 [0.569–0.677]	0.617 [0.565–0.669]	0.614 [0.559–0.666]	0.563 [0.507–0.618]	0.543 [0.480–0.601]
SAPS II	0.632 [0.575–0.687]	0.630 [0.573–0.681]	0.625 [0.566–0.679]	0.582 [0.520–0.636]	0.577 [0.515–0.635]

EASIX—Endothelial Activation and Stress Index; SAPS II—Simplified Acute Physiology Score II; APACHE II—Acute Physiology and Chronic Health Evaluation II; SOFA—Sequential Organ Failure Assessment.

## Data Availability

The data presented in this study are available in Figshare at https://doi.org/10.6084/m9.figshare.31807600.

## Abbreviations

The following abbreviations are used in this manuscript:

APACHE II	Acute Physiology and Chronic Health Evaluation II
CI	Confidence Interval
CRP	C-reactive protein
EASIX	Endothelial Activation and Stress Index
ICU	Intensive Care Unit
IQR	Interquartile Range
LDH	Lactate dehydrogenase
mEASIX	Modified Endothelial Activation and Stress Index
OR	Odds Ratio
PCT	Procalcitonin
SAPS II	Simplified Acute Physiology Score II
sEASIX	Simplified Endothelial Activation and Stress Index
SOFA	Sequential Organ Failure Assessment
